# Unlocking Precision in Spinal Surgery: Evaluating the Impact of Neuronavigation Systems

**DOI:** 10.3390/diagnostics14161712

**Published:** 2024-08-07

**Authors:** Mehmet Yigit Akgun, Mete Manici, Ozkan Ates, Melis Gokdemir, Caner Gunerbuyuk, Mehmet Ali Tepebasili, Oguz Baran, Turgut Akgul, Tunc Oktenoglu, Mehdi Sasani, Ali Fahir Ozer

**Affiliations:** 1 Department of Neurosurgery, Koc University Hospital, 34010 Istanbul, Turkey; mehmetalitepebasili@gmail.com (M.A.T.); oguzbaran@gmail.com (O.B.); tuncoktenoglu@gmail.com (T.O.); sasanim@gmail.com (M.S.); alifahirozer@gmail.com (A.F.O.); 2 Spine Center, Koc University Hospital, 34010 Istanbul, Turkey; drcaner@yahoo.com (C.G.); trgtakgul@gmail.com (T.A.); 3 Department of Anesthesiology and Reanimation, Koc University Hospital, 34010 Istanbul, Turkey; mmanici@kuh.ku.edu.tr; 4 Medical Faculty, Sapienza University of Rome, 00185 Rome, Italy; mgokdemir16@ku.edu.tr; 5 Department of Orthopaedics and Traumatology, Koc University Hospital, 34010 Istanbul, Turkey

**Keywords:** O-arm, neuronavigation, instrumentation, spine, surgery

## Abstract

Objective: In spine surgery, ensuring the safety of vital structures is crucial, and various instruments contribute to the surgeon’s confidence. This study aims to present outcomes from spinal cases operated on using the freehand technique and neuronavigation with an O-arm in our clinic. Additionally, we investigate the impact of surgical experience on outcomes by comparing early and late cases operated on with neuronavigation. Method: We conducted a retrospective analysis of spinal patients operated on with the freehand technique and neuronavigation in our clinic between 2019 and 2020, with a minimum follow-up of 2 years. Cases operated on with neuronavigation using the O-arm were categorized into early and late groups. Results: This study included 193 patients, with 110 undergoing the freehand technique and 83 operated on utilizing O-arm navigation. The first 40 cases with neuronavigation formed the early group, and the subsequent 43 cases comprised the late group. The mean clinical follow-up was 29.7 months. In the O-arm/navigation group, 796 (99%) of 805 pedicle screws were in an acceptable position, while the freehand group had 999 (89.5%) of 1117 pedicle screws without damage. This rate was 98% in the early neuronavigation group and 99.5% in the late neuronavigation group. Conclusions: The use of O-arm/navigation facilitates overcoming anatomical difficulties, leading to significant reductions in screw malposition and complication rates. Furthermore, increased experience correlates with decreased surgical failure rates.

## 1. Introduction

Back pain is a pervasive issue worldwide, affecting a substantial portion of the population regardless of geographical location. As life expectancy increases and populations age, the incidence of back pain and degenerative spinal conditions is expected to escalate, posing significant medical and socioeconomic challenges. In addition to degenerative processes, spinal instability can arise from various factors such as trauma, inflammation, or tumors, often necessitating surgical intervention to alleviate symptoms and restore function.

The primary objective of spinal surgery is to address spinal instability and associated symptoms by restoring the spine’s structural integrity and function. This typically involves procedures aimed at stabilizing the spinal column and relieving pressure on affected nerves. Achieving optimal outcomes in spinal surgery requires a meticulous approach, with surgeons aiming for precise instrumentation placement to avoid complications such as neurological deficits or vascular injury. Advanced imaging technologies, including intraoperative navigation systems and robotic assistance, have enhanced surgical precision and contributed to improved patient outcomes.

Ultimately, the goal of spinal surgery is to improve patient’s quality of life by alleviating pain, restoring mobility, and minimizing the risk of future complications. As our understanding of spinal conditions continues to evolve and technological advancements in surgical techniques progress, the outlook for individuals suffering from spinal instability and related symptoms is increasingly promising. However, ensuring the success of spinal surgery requires careful patient selection, thorough preoperative planning, and skilled execution in the operating room, emphasizing the importance of a multidisciplinary approach to spinal care.

Pedicle screw insertion is a critical procedure in spine instrumentation due to its proximity to vital structures such as neurovascular components, the spinal cord, and nerve roots. This operation can be particularly challenging given the diverse structures and positions of the spine [1]. Correct placement of the pedicle screw is crucial in order to avoid neurological injuries, penetration of the lateral cortical walls, and damage to important structures in the anterior neighborhood. Precision in this operation helps reduce the need for revision surgeries and enhances postoperative patient comfort. Consequently, there has been a gradual increase in the utilization of O-arm intraoperative tomography-assisted neuronavigation systems, in conjunction with anatomical signs observed using C-arm fluoroscopy, as an alternative to the traditional “freehand” method of pedicle screw placement [2,3].

In contrast to intraoperative fluoroscopy, navigation systems offer 3D mapping of the patient’s anatomical structure, providing significant convenience in surgical planning. Effective and accurate planning is crucial for achieving satisfactory results post-surgery. Navigation systems find applications in various areas of spinal surgery, encompassing degenerative spine conditions, deformity surgery, and traumatic cases [4]. Previous studies have highlighted the advantages of O-arm navigation systems in improving the accuracy of pedicle screw placement and reducing the risk of complications in spinal surgery. However, there is a need for comprehensive assessments that extend beyond immediate postoperative outcomes and encompass long-term clinical results across different regions of the spine.

This article aims to assess the effectiveness of the O-arm navigation system, explore the impact of experience on outcomes, and compare the O-arm system with the freehand technique in cases stabilized by the transpedicular screw method. In addition, we tried to demonstrate the learning curve of the O-arm/navigation technique by comparing our early and late cases. Importantly, this evaluation extends beyond a single region, encompassing the entire spinal column (cervical, thoracic, and lumbar). The focus is not only on the immediate postoperative accuracy of pedicle positioning, as commonly found in the literature, but also on the long-term clinical outcomes of patients undergoing navigation-assisted surgery. Additionally, this article aims to emphasize the various stages of navigation and potential pitfalls, highlighting how navigation can be employed comprehensively and accurately throughout the entire surgical procedure.

## 2. Materials and Methods

### 2.1. Ethical Standards

All procedures in this study were conducted in accordance with the ethical standards of the institutional and national research committee and adhered to the principles outlined in the 1964 Helsinki Declaration and its later amendments or comparable ethical standards. Informed consent was obtained from all participants included in the study prior to their involvement.

### 2.2. Patient Selection

Spinal patients who underwent surgery at our clinic between 2019 and 2021 were retrospectively analyzed. Inclusion criteria comprised patients with a minimum of 2 years of clinical and radiological follow-up. Cases operated on using both the freehand technique and O-arm (Medtronic PLC, Littleton, MA, USA) with neuronavigation systems (Medtronic) were included. In our analysis, we stratified the data into several groups to ensure a comprehensive evaluation of the neuronavigation system’s impact on spinal surgery outcomes. The primary grouping involved comparing early cases (first 40) and late cases (subsequent 43) utilizing neuronavigation, while secondary groupings categorized patients based on the anatomical region of surgery (cervical, thoracic, lumbar). Additionally, we differentiated between surgeries performed with freehand techniques (110 patients) and those using O-arm navigation (83 patients).

### 2.3. Imaging and Assessment

Patients underwent detailed neurological and radiological imaging examinations for pain localization diagnosis. This included direct radiography, computed tomography (CT), and magnetic resonance imaging (MRI). The qualitative assessment of screw positioning utilized the 6-grade scoring system described by Zdichavsky et al., where Grade Ia represents an excellent position [5]. In addition to intraoperative O-arm extraction for control purposes, standard control CT scans were conducted on the first day after completing the procedure.

### 2.4. Clinical Evaluation

Patients were categorized into three groups based on pain locations: cervical, thoracic, and lumbar. Demographic data, pathologies leading to the operation, surgical complications, screw malposition rates, perioperative operation data, and clinical and radiological follow-ups of the operated-on cases were examined. Pre-operative Visual Analogue Scale (VAS) and Oswestry Disability Index (ODI) scores for each patient were compared with scores calculated during postoperative routine controls.

### 2.5. Patient Follow-Up

Each patient’s medical history, recorded during routine clinical follow-ups, was compared with initial complaints and analyzed. Patients were examined for improvement through both physical examination and symptoms compared to the preoperative period, and those whose clinical symptoms improved were documented as benefiting from the operation.

### 2.6. Surgical Technique

For cases operated on with the freehand technique, the standard midline posterior approach to the spine was used under general anesthesia with endotracheal intubation. The vertebral body was then exposed and, after exposing the levels of pathology, stabilization was performed according to the anatomical region using the standard freehand technique under C-arm fluoroscopy guidance.

**Figure 1:**
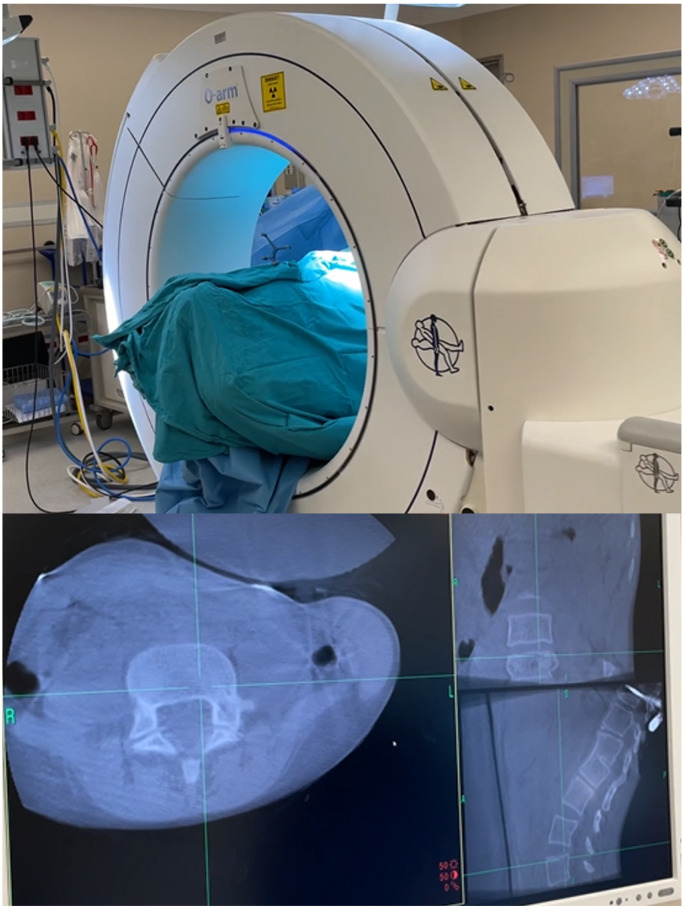
With the O-arm, a CT scan of the relevant area is taken. Planning can be done based on the CT images.

**Figure 2:**
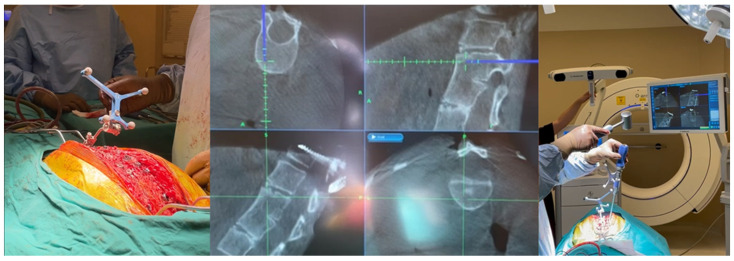
Placing the passive frame, checking the orientation and angle of the screw, and tapping with the help of a hammer.

**Figure 3:**
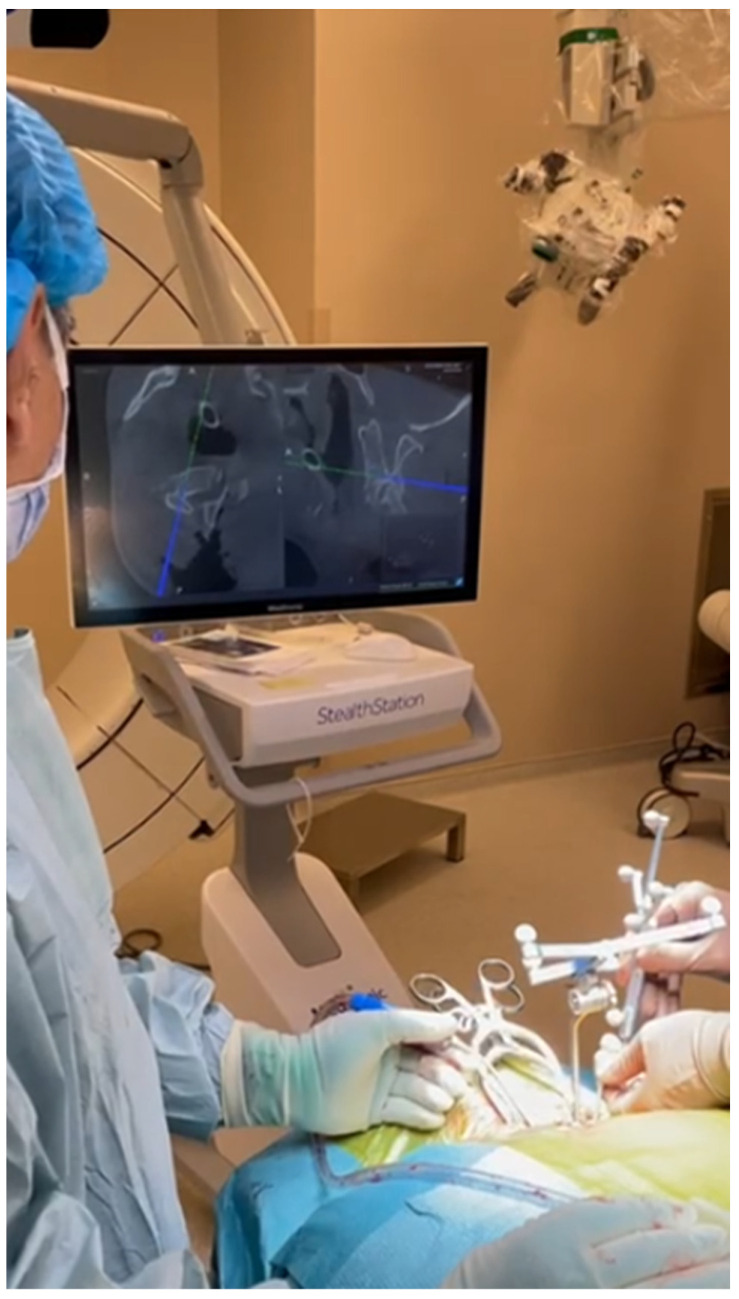
Another view of the placing pedicle screw with navigation systems.

For neuronavigation cases, preparations were made for easy imaging by the O-arm, and both the patient and the anesthesia team were positioned appropriately. The use of a carbon fiber operation table and skull clamp helped prevent artifacts and ensured quality images. After the bilateral paravertebral muscles were dissected, the “passive frame” used for navigation registration was attached to the spinous process near the operation area. Care was taken to securely place the frame without breaking the spinous process during this procedure. It was also emphasized not to touch the frame during the operation. Anesthesia was deepened as much as possible from this stage to prevent any patient movement. After the O-arm images were transferred to the navigation system, the instruments to be used were verified. Tapping with the help of a hammer or high-speed drill was performed to prevent patient movement and ensure correct imaging in the navigation system. Additionally, the accuracy of anatomical landmarks was checked by navigation to verify that the instruments indicated the correct location. Instant revision was facilitated by the O-arm images after the stabilization process. The steps of the surgical technique are shown in Figure 1, Figure 2 and Figure 3 and Supplementary Video S1.

### 2.7. Statistical Analysis

All statistical analyses were performed using IBM SPSS 20.0 software (IBM Corp., Armonk, NY, USA). Data were reported as mean ± SD for normally distributed continuous variables and as number and percentage for dichotomous variables. Data were compared using the chi-square test for categorical data and the *t*-test or analysis of variance for continuous data. A two-tailed *p* < 0.05 was considered to indicate significant differences.

## 3. Results

A total of 193 patients, comprising 110 operated on with the freehand technique and 83 with the O-arm navigation, were included in this study. Among the patients, 90 were female and 103 were male, with a mean age of 59.6 years ± 13.7 years. The mean clinical follow-up was 29.7 months (range 24–40 months). The baseline demographic and procedural characteristics by localization are summarized in Table 1.

When analyzing surgical pathologies, it was found that 119 patients underwent surgery for degenerative reasons (disc hernia, spondylolisthesis, stenosis), 35 for deformity, 24 for oncological reasons, and 15 for traumatic conditions (Table 2). In terms of region distribution, 42 patients were operated on in the cervical region, 47 in the cervicothoracic region, 40 in the thoracic region, and 64 in the thoracolumbar region. A total of 57 patients (29.5%) underwent short-segment stabilization (2 or fewer levels), while 136 patients (70.5%) underwent long-segment stabilization (more than 2 levels) (Table 1 and Table 2).

In the O-arm navigation group, 796 (99%) of 805 pedicle screws were in an acceptable position, while in the freehand group, 999 (89.5%) of 1117 pedicle screws met the criteria and were without cortical wall penetration or damage to adjacent organs. Excellent positions (Ia) were observed in 788 screws (97.7%) in the initial intraoperative imaging with O-arm navigation, compared to 948 screws (84.9%) showing an excellent position (Ia) on postoperative day one CT in the freehand group. The rates for the early and late O-arm navigation groups were 96.9% and 98.5%, respectively. A total of eight screws had complications, including six with lateral penetration (4-IIa, 2-IIIa) and two with medial penetration (1-IIb, 1-IIIb), all detected and revised perioperatively. In the freehand group, 82 screws had lateral penetration (77-IIa, 5-IIIa) and 36 had medial penetration (29-IIb, 7-IIIb) on postoperative day one CT. Three patients underwent revision surgery in the freehand group (two for medial penetration, one for lateral penetration) (*p* < 0.05). No complications or additional deficits occurred due to screw positions in the O-arm navigation group, whereas revision surgery was performed in one patient in the freehand group due to neurological deficit. Superficial wound infection was observed in five patients (two in the neuronavigation group, three in the freehand group), and CSF leakage was observed in one patient (freehand group).

At the 3-month follow-up, a statistically significant decrease in VAS and ODI scores was observed in all patients (*p* < 0.05) (Table 3). The operation times and surgical information for the late/early group are presented in Table 4, Table 5 and Table 6. In routine patient follow-ups, significant clinical relief was observed, with no complaints of local pain. The stability, confirmed subjectively by the absence of axial pain and objectively by CT findings and dynamic radiographs, showed no implant-related complications requiring revision at the last follow-up.

## 4. Discussion

The rapid expansion of spinal surgery is a testament to both the advancements in medical technology and the increasing prevalence of spinal conditions, particularly among aging populations. Improved imaging diagnostics have transformed the approach to spinal procedures, facilitating precise diagnoses and targeted treatments. Furthermore, the introduction of new techniques and tools has broadened the capabilities of spinal surgeons, allowing them to effectively address a wider array of conditions.

Despite these advancements, the reliance on intraoperative imaging, often utilizing ionizing radiation, raises significant concerns due to the associated health risks. While such imaging modalities greatly assist surgeons in visualizing the complex structures of the spine during surgery, they also pose potential risks to both patients and surgical staff.

To address these concerns, efforts are underway to optimize imaging protocols to minimize radiation exposure while maintaining diagnostic quality. Additionally, advancements in imaging technology, including the development of low-dose techniques and the utilization of alternative modalities such as intraoperative MRI or CT scans, aim to reduce radiation exposure without compromising surgical outcomes.

As spinal surgery continues to progress, it is crucial to prioritize patient safety by carefully weighing the benefits of intraoperative imaging against the risks of radiation exposure. Exploring alternative imaging strategies and continuing to refine existing techniques will further enhance patient care in this evolving field. The challenge of pedicle screw insertion further underscores the importance of safe and precise intraoperative imaging in spinal surgery. The improper placement of pedicle screws can lead to a range of complications, including nerve and dural injuries, persistent pain, neurological deficits, CSF fistulas, infections, and increased costs for patients. Efforts to enhance imaging guidance during pedicle screw insertion, such as the use of navigation systems and robotic assistance, aim to improve the accuracy of this operation and minimize complications, emphasizing the critical role of intraoperative imaging in ensuring successful surgical outcomes.

Frameless stereotactic navigation was first reported for use in spine surgery in 1999, specifically for pedicle screw insertion in the lumbar region [6]. Its prevalence increased as spine surgeries transitioned to more minimally invasive procedures (MIS), driven by its perceived ability to enhance accuracy and the growing availability of navigation systems. Unlike freehand procedures that rely on anatomical landmarks and intraoperative fluoroscopy, stereotactic navigation provides three-dimensional imaging, improving understanding of the precise placement during surgery. The most commonly used stereotactic navigation instruments, in order of advancement, include the Stealth Station (Medtronic, Memphis, Tennessee, USA), O-arm (Medtronic, Dublin, Ireland), and O-arm II [7].

One of the main barriers to the usage of intraoperative navigation is the high acquisition and maintenance costs. However, it should be noted that secondary costs related to screw malposition and revision surgery create a higher economic burden after operations performed with the classical freehand method. According to Dea et al. [8], the use of intraoperative navigation in spinal surgery has the potential for significant cost-effectiveness. Within one year of surgery, 2 patients operated on with navigation required revision surgery, as opposed to 15 patients with the freehand technique. This difference in the requirement for revision surgery was statistically significant, with *p* = 0.014. In our series, revision surgery was not required for any of the patients operated on with neuronavigation using O-arm in the postoperative period. The reduction in discharge times, along with the decrease in postoperative complications, are factors contributing positively to cost-effectiveness.

Repeated ionizing radiation exposure produced by fluoroscopy has been a significant concern for every surgical team. One of the advantages of neuronavigation systems compared to fluoroscopy is the reduced radiation exposure. Bratschitsch et al. [9] reported that the use of O-arm navigation, compared to C-arm fluoroscopy, significantly lowered radiation exposure during lumbosacral spinal fusion procedures. The trial concluded that a spinal surgeon could perform 10,000 surgeries with O-arm compared to 883 surgeries with C-arm before reaching the maximum permissible annual effective radiation dose. Continuous exposure to radiation with the classical method, accompanied by C-arm fluoroscopy, is one of the negative aspects of the procedure. With the O-arm, a total of two tomography scans are performed intraoperatively, for the purpose of navigation registration and for the control of screw placements, thus reducing exposure to a significant extent.

In the literature, the effects of navigation use in spine surgery on the operation time are among the topics discussed. Due to the setup time and the intraoperative imaging duration, many assume that the operational time is increased. Khanna et al. [10], in 2016, retrospectively observed 63 freehand and 70 navigation-guided 1-level lumbar transpedicular instrumentation operations and recorded the total operation time for the cases. Contrary to the popular assumption in the literature, the data showed a significantly shorter duration for navigation-guided surgeries with 4:30+/−0:42 h for navigation vs. 4:53+/−0:39 h for freehand operations (*p* = 0.0013). On the other hand, Balling et al. [11] reported in 2018 that the use of navigation during pedicle screw instrumentation increased the operation time by 13 min compared to C-arm usage (*p* = 0.81). There are several conflicting results on the effects of navigation on the operation duration, and the literature is not in consensus. From our sample, we observed that the operation time was slightly increased due to the additional setup time of the O-arm. When we compared the operation times in both groups, we found 253 ± 38 min in the navigation group and 226 ± 26 min in the freehand group. Although the operation time was longer in the neuronavigation group, this difference was not statistically significant. Furthermore, when we examined the early and late neuronavigation groups, we found that the operation time was 276 ± 56 min in the early group and 230 ± 18 min in the late group. Moreover, we concluded that the duration of the operation can be reduced with experience. The operation times of the late neuronavigation group and the freehand group were observed to be almost the same.

Pedicle screw insertion can present challenges due to the variation in the anatomical structure of the spine, particularly the complex anatomy of the cervical and thoracic regions. Deformities such as scoliosis, kyphosis, and degenerative changes in the spine can cause angular, rotational, and coronal excursions, resulting in difficulties with accurate pedicle screw insertions. In the literature, these challenges can manifest as increased stabilization complications, deficits, and revision surgeries [12,13]. One way to address these challenges is the use of intraoperative navigation systems.

Feng et al. [14] conducted a systematic review in 2020 of articles comparing the accuracy levels of O-arm navigation with those of C-arm fluoroscopy in pedicle screw insertion in spinal surgical cases. They evaluated screw positioning postoperatively and concluded that correct insertion significantly increased with the usage of O-arm intraoperative navigation. In our series, we achieved high accuracy rates in screw placement throughout the entire spinal column with navigation and O-arm. We observed significantly lower rates of malposition and intraoperative screw revision in the neuronavigation group. The rate of screws in an unacceptable position was 10.5% in the freehand group and 1% in the neuronavigation group. In the freehand group, three cases underwent revision surgery, while no revision surgery was performed in the neuronavigation group. Moreover, our malposition and revision rates were even lower compared to those in the literature [15,16]. In parallel with the literature, there was a significant difference in the success rate of the late neuronavigation group compared to the early group [17]. While the rate of screws in an excellent position (Ia) was 96.9% in the early group, it was 98.5% in the late group.

Although there are varying opinions in the literature about the significant benefits of using navigation, its importance is undisputed, especially in minimally invasive spine surgery, deformity correction surgery, and revision cases where anatomical landmarks are impaired [18,19,20]. However, maintaining navigational accuracy during surgery is one of the most critical issues. The stability of the frame placed, the use of a carbon fiber table during the operation, ensuring a sufficient depth of anesthesia, and keeping the patient as stable as possible during the tapping phase are crucial steps. Paying attention to these considerations will automatically increase the rate of favorable results with navigation. In the clinical follow-up of our patients, significant improvements in neurological functions, low complication rates, and the absence of revision surgery are directly related to the correct use of navigation.

Any surgical method has a learning curve, and this may be reflected in the correlation between more surgical experience and better patient outcomes. Since the neuronavigation system is a new technique and involves a training process, some surgeons may hesitate to use these procedures. Wood et al. observed that, as familiarity with the neuronavigation system increased, the frequency of screw malposition and intraoperative positive EMG signals significantly decreased [17]. In the present study, we also observed a significant improvement in operative time and successful screw position rates in parallel with the increasing experience of the surgeon. The defined rates of problems may continue to decline, falling below those reported in earlier publications, as surgeons gain experience in these techniques.

### 4.1. Future Research Directions

Future research could focus on further optimizing the use of O-arm navigation systems in spine surgery by exploring advanced techniques and technologies. This may involve the integration of artificial intelligence algorithms to improve preoperative planning and intraoperative navigation, as well as the development of new imaging modalities for enhanced visualization of spinal anatomy. Additionally, longitudinal studies could investigate the long-term durability and stability of pedicle screw fixation guided by O-arm navigation, providing valuable insights into the sustainability of outcomes over time.

### 4.2. Possible Applications of the Research

The findings of this study have the potential to inform clinical practice and guide decision-making in spine surgery. By demonstrating the effectiveness of O-arm navigation systems in improving pedicle screw placement accuracy and patient outcomes, the research may encourage wider adoption of these technologies in surgical settings. Additionally, the insights gained from this study could contribute to the development of evidence-based guidelines for the use of navigation systems in different spinal pathologies and surgical procedures, ultimately improving the quality of care for patients undergoing spinal surgery.

## 5. Limitations

The retrospective design of this study is one of its most important limitations. Prospective randomized controlled trials and sample enlargement will yield more valuable results. Given the retrospective nature of this study, forming the control group was challenging.

## 6. Conclusions

Great improvements have been achieved in developing better techniques and tools in spine surgery. It is envisaged that progress in the four focus areas discussed will lead to better outcomes and reduced burdens on the future of both our patients and the health care system. Neuronavigation enhances the accuracy of pedicle screw placement in deformed spines, reducing the rate of misplaced screws and potential complications. Since neuronavigation is a new technique, a training process is required, as with any surgical technique. However, it seems that, as experience in neuronavigation increases, successful surgical outcome rates also increase.

## Figures and Tables

**Table 1:** Summarized Data of Patients.

		Mean ± SD	Median (IQR)
Age (years)		59.6 ± 13.7	66.0 (49.0–83.0)
Clinical and Radiological Follow Up (month)		29.7 ± 4.3	
Duration of Clinical Symptoms (month)		4.7 ± 1.1	
		n	%
Gender	Female	90	46.6
	Male	103	53.4
Stabilization	Short segment	57	29.5
	Long segment	136	70.5
Localization	Cervical; Cervicothoracic	42; 47	21.7; 24.3
	Thoracic; Thoracolumbar	40; 64	20.7; 33.3

**Table 2:** Indications for Surgery and Screw Positions.

**Neuronavigation**				
	**Deformity**	**Tumor**	**Degenerative**	**Trauma**
Cervical	3	4	9	2
Cervicothoracic	5	2	12	1
Thoracic	6	3	9	1
Thoracolumbar	2	2	20	2
Screw Malpositions	2-IIa, 1-IIIa	1-IIb	2-IIa, 1-IIIb, 1-IIIa	
**Freehand Technique**				
	**Deformity**	**Tumor**	**Degenerative**	**Trauma**
Cervical	5	4	11	4
Cervicothoracic	4	4	17	2
Thoracic	4	3	13	1
Thoracolumbar	6	2	28	2
Screw Malpositions	19-IIa, 2-IIIb, 1-IIIa, 9-IIb	4-IIa,1-IIb	54-IIa, 5-IIIb, 3-IIIa, 19-IIb	1-IIIa

**Table 3:** Comparison of VAS And ODI Scores of Patients Before and After Treatment.

		Preoperative		Postoperative		Change		*p* *
		Mean ± SD	Median (IQR)	Mean ± SD	Median (IQR)	Mean ± SD	Median (IQR)	
Neuronavigation	VAS score	8.5 ± 0.6	8.0 (8.0–9.0)	3.3 ± 0.8	3.0 (3.0–3.0)	5.2 ± 1.2	5.0 (5.0–6.0)	0.006
	ODI score	65.2 ± 15.4	66.0 (56.0–72.0)	23.9 ± 11.4	20.0 (16.0–24.0)	41.3 ± 15.0	44.0 (34.0–48.0)	0.007
Freehand	VAS score	8.2 ± 0.4	8.1 (8.0–9.0)	3.6 ± 0.9	3.1 (3.0–3.3)	4.6 ± 1.2	5.2 (5.0–6.3)	0.008
	ODI score	64.1 ± 16.6	67.0 (55.0–73.0)	24.3 ± 12.1	21.0 (17.0–23.0)	39.8 ± 15.9	43.0 (33.0–47.0)	0.009

* Repeated measure ANOVA analysis was applied.

**Table 4:** The Surgical Information of Early Group.

Neuronavigation Early Group (n:40)				
	Deformity	Tumor	Degenerative	Trauma
Cervical	-	2	5	-
Cervicothoracic	1	2	7	1
Thoracic	3	1	4	1
Thoracolumbar	3	-	10	-
Screw Malpositions	2-IIa, 1-IIIa	1-IIb	1-IIa, 1-IIIb, 1-IIIa	

**Table 5:** The Surgical Information of Late Group.

Neuronavigation Late Group (n:43)				
	Deformity	Tumor	Degenerative	Trauma
Cervical	2	4	4	1
Cervicothoracic	1	1	5	2
Thoracic	4	-	5	1
Thoracolumbar	2	1	10	-
Screw Malpositions			1-IIa	

**Table 6:** The Comparison of Operation Times.

	Operation Time (Min)
Freehand	226 ± 26
Neuronavigation -Early Group -Late Group	253 ± 38
	276 ± 56
	230 ± 18

## Data Availability

The datasets used and/or analyzed during the current study are available from the corresponding author upon reasonable request.

## Supplementary Materials

The following supporting information can be downloaded at: https://www.mdpi.com/article/10.3390/diagnostics14161712/s1, Video S1: The Stages of Neuronavigation system.
